# Targeted ORF8-N Sanger Sequencing as a SARS-CoV-2 Surveillance Contingency During Supply Shortages

**DOI:** 10.1590/0037-8682-0591-2025

**Published:** 2026-09-14

**Authors:** Brenno Vinícius Martins Henrique, Helder Andrey Rocha Gomes, Marina da Costa Ribeiro, Bruna Cândido Guido, Ricardo Camargo, Lucas Luiz Vieira, Mariana Matos Roll, Jordan Barros da Silva, Glaura Regina de Castro e Caldo Lima, Fabiano José Queiroz Costa, Agenor de Castro Moreira dos Santos

**Affiliations:** 1 Laboratório Central de Saúde Pública do Distrito Federal, Brasília, DF, Brasil.; 2 Hospital da Criança de Brasília José Alencar, Brasília, DF, Brazil.; 3 Centro Universitário do Distrito Federal, Brasília, DF, Brasil.

**Keywords:** Genomic, Sequence analysis, Pandemics, Public health, Supply shortages

## Abstract

**Background:**

Global shortages of next-generation sequencing (NGS) reagents threatened SARS-CoV-2 genomic surveillance in low- and middle-income countries during the COVID-19 pandemic.

**Methods:**

During the 2021 NGS reagent shortages, we implemented targeted ORF8-N Sanger sequencing for SARS-CoV-2 variant surveillance in Brazilian public health laboratories.

**Results:**

*In silico* analysis of whole-genome sequencing (WGS)-derived SARS-CoV-2 genomes from the Federal District, Brazil, showed that the ORF8-N target discriminated the major 2021 lineages (Gamma and Delta) and enabled analysis of ˃300 samples despite constrained NGS access.

**Conclusions:**

Targeted ORF8-N Sanger sequencing was a useful temporary contingency during NGS reagent shortages but offered lower phylogenetic resolution than WGS.

Global strategies for genomic surveillance have become increasingly important in detecting, monitoring, and controlling infectious diseases, particularly during the coronavirus disease 2019 (COVID-19) pandemic caused by severe acute respiratory syndrome coronavirus 2 (SARS-CoV-2). Genomic surveillance systems enable real-time tracking of viral evolution, identification of emerging variants, and assessment of vaccine efficacy^1^. These data support public health decision-making and facilitate timely interventions. However, the capacity to perform effective genomic surveillance varies substantially across countries. Low- and middle-income countries often face interruptions in reagent availability, limited financial resources, insufficient infrastructure, and a lack of skilled personnel to generate and analyze genomic data, all of which can create surveillance “dark spots.” Despite these obstacles, the COVID-19 pandemic demonstrated the need to integrate genomic surveillance into global health strategies, highlighting the importance of sustained investment and international support to build resilient surveillance systems worldwide^2^.

Although whole-genome sequencing (WGS) remains the gold standard, the COVID-19 pandemic exposed the fragility of relying solely on complex supply chains. While not as comprehensive as WGS, alternative techniques offer important advantages in terms of cost and accessibility. For example, polymerase chain reaction (PCR)-based assays can rapidly discriminate variants of concern by targeted gene deletions^3^. Despite its lower suitability for high-throughput applications, Sanger sequencing has proven valuable for the rapid detection and tracking of specific mutations within the SARS-CoV-2 spike gene, making it a practical tool in resource-limited settings^4^. However, limitations include lower throughput, potential sampling and sequencing errors, and the inability to provide the complete genomic context required for in-depth evolutionary analyses^5^.

The emergence and global spread of SARS-CoV-2 variants posed considerable public health challenges, driven by the high mutation rate of RNA viruses, which contributed to changes in virulence and antigenic escape^6^. These mutations were predominantly identified in the ORF1a, ORF1b, spike (S), and nucleocapsid (N) genomic regions^7^ ^,^ ^8^. Although WGS remained the gold standard for mutation tracking, it was costly and technically demanding^4^. In this context, specifically during the 2021 supply crisis, our group faced a critical decision: halt surveillance or adapt the available resources. We implemented a Sanger sequencing-based contingency plan to bridge genomic surveillance between the Central Public Health Laboratory of the Federal District (Lacen-DF) and the Translational Research Laboratory of a pediatric hospital, both part of Brazil’s Unified Health System. 

Less than 0.2% of samples in Brazil had been sequenced by August 2021, despite the country’s high number of cases and deaths^9^ ^-^ ^10^. Thus, we designed primers flanking the region between the ORF8 and N genes (forward primer: GCTGGTTCTAAATCACCCATTC; reverse primer: GGGTGCATTTCGCTGATTT) to sustain surveillance capacity despite the next-generation sequencing (NGS) blackout. The resulting amplicon, spanning 28086-28294 of the SARS-CoV-2 reference genome (NC_045512.2), enabled discrimination of the variants prevalent at that time.

Nasopharyngeal specimens that tested positive for SARS-CoV-2 by routine reverse transcription-quantitative PCR (RT-qPCR) were considered for the contingency sequencing workflow if residual material was available and the Ct value was within the operational amplification range established for the assay. The Ct value guided sequencing allocation but was not used as a formally validated analytical sensitivity threshold. The ORF8-N target demonstrated improved detectability under the evaluated operational conditions, potentially owing to the shorter amplicon size and the presence of subgenomic RNA (sgRNA) transcripts associated with viral transcriptional activity^11^. RT-qPCR cycle threshold (Ct) values were used to guide sequencing of both the ORF8-N and spike targets^11^ ^,^ ^12^ (Ct < 26) or preferential sequencing of the ORF8-N target (Ct > 26) (Figure 1A), thereby improving sequencing success in samples with lower viral RNA concentrations.

**FIGURE 1:**
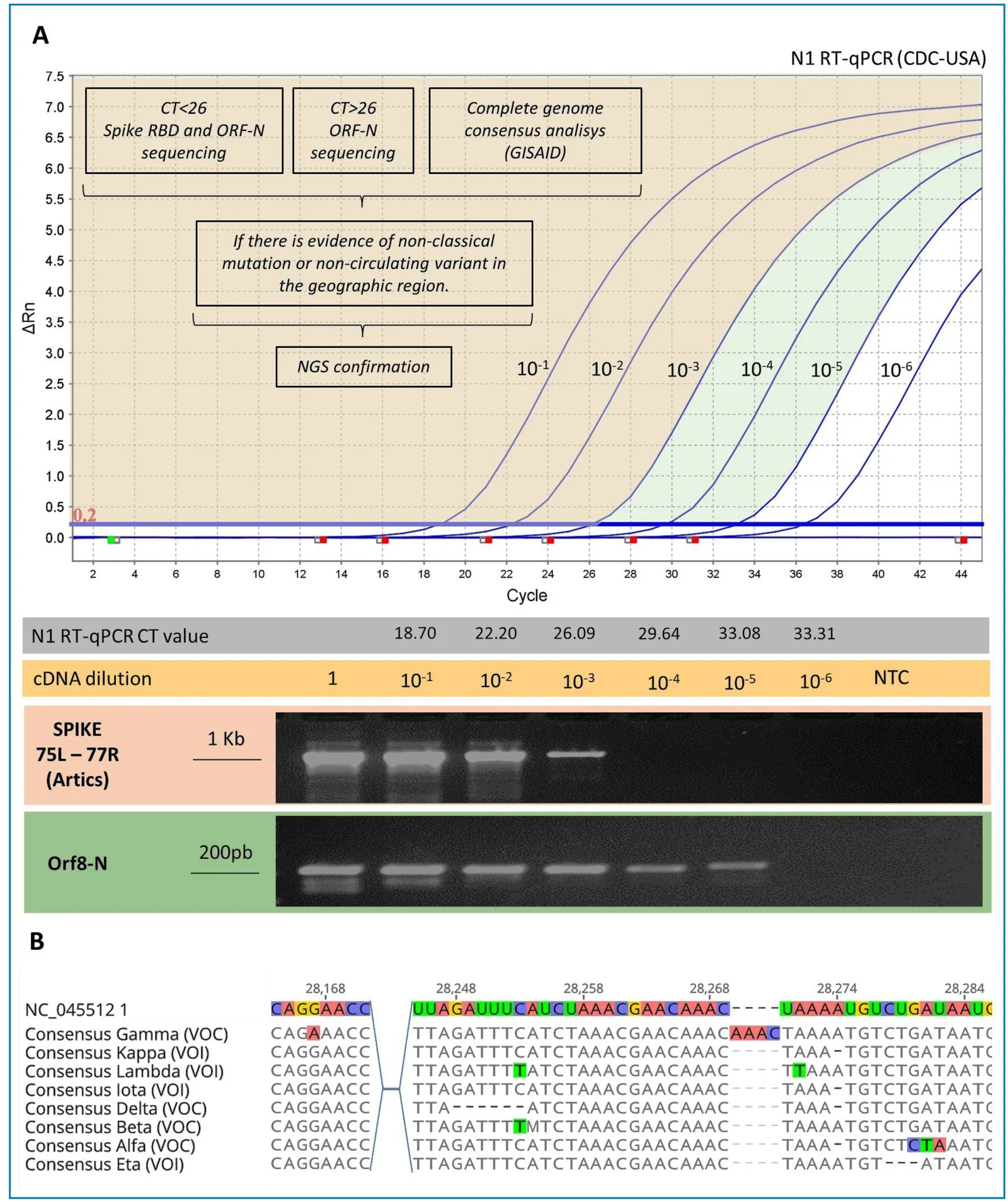
Sequencing workflow based on the Orf8-N intergenic region. **(A)** Assay amplification performance assessed by reverse transcription-quantitative PCR (RT-qPCR) for detection of the N gene (N1; USA-CDC protocol) and the spike receptor-binding domain (RBD) domain and the Orf8-N targets, showing improved detectability of the ORF8-N target. Assays were performed using serial dilutions of a positive sample. After diagnosis, routine samples were selected for Sanger sequencing according to RT-qPCR Ct values. NTC, negative test control. **(B)** SARS-CoV-2 lineage discrimination based on mutations observed in the ORF8-N region. Consensus sequences were generated from complete SARS-CoV-2 genomes available in GISAID.

To assess the discriminatory capacity of the ORF8-N target, we performed an *in silico* analysis using complete SARS-CoV-2 genomes deposited in GISAID. The ORF8-N region corresponding to the Sanger amplicon was extracted from WGS-derived genomes and compared with lineage assignments based on the corresponding whole-genome sequences. This analysis evaluated whether the restricted ORF8-N target retained sufficient lineage-discriminatory information for the variants circulating during the evaluated epidemiological period, rather than estimating the analytical sensitivity or specificity of the complete Sanger workflow. 

In addition to the operational Sanger implementation, we assessed ORF8-N discriminatory performance using WGS-derived sequences from GISAID. The extracted ORF8-N region was analyzed together with lineage reference sequences to evaluate whether the sequences clustered within the expected lineage clades. Our analysis included 6,136 Brazilian SARS-CoV-2 sequences representing the Alpha, Beta, Gamma, Delta, and Lambda variants, and 8,000 global sequences representing additional variants, including Kappa, Iota, and Eta (Figure 1B). Notably, all sequences assigned to the same lineage consistently displayed the characteristic molecular signature illustrated in Figure 1B. Lineage discrimination and nomenclature followed the Pango lineage classification system.

The Gamma variant, first detected in Brazil, was characterized by a G>A substitution at nucleotide 28167 and a 4-nucleotide insertion (28258_28262insAAAC) between ORF8 and the N gene. The Delta variant, first identified in India, had a 6-nucleotide deletion (28236_28241del) in ORF8. Additional variants, including Alpha and Beta, were also differentiated using this approach. We also performed partial sequencing using ARTIC primer pair 75L/77R to identify variants defined by key spike mutations, including S340V, K417T, E484K, and N501Y.

Additionally, complete SARS-CoV-2 genomes from the Federal District, Brazil, deposited in GISAID in 2021 were analyzed^13^ to assess the discriminatory power of the selected genomic region between the ORF8 and N genes. The target region was extracted from each genome, and a maximum likelihood phylogenetic tree was constructed using IQ-TREE. Samples were interpreted based on their clustering with the corresponding lineage reference branch and, when available, the presence of lineage-informative mutations within the amplified region. Within the 2021 epidemiological context of the Federal District, the ORF8-N region retained sufficient discriminatory information to separate the main circulating lineages (Figure 2 and Supplementary Table 1). Notably, all SARS-CoV-2 genomes generated exhibited the same lineage-specific clustering pattern as the reference dataset, consistently grouping with their respective lineage clades and displaying the corresponding ORF8-N molecular signatures.

**FIGURE 2:**
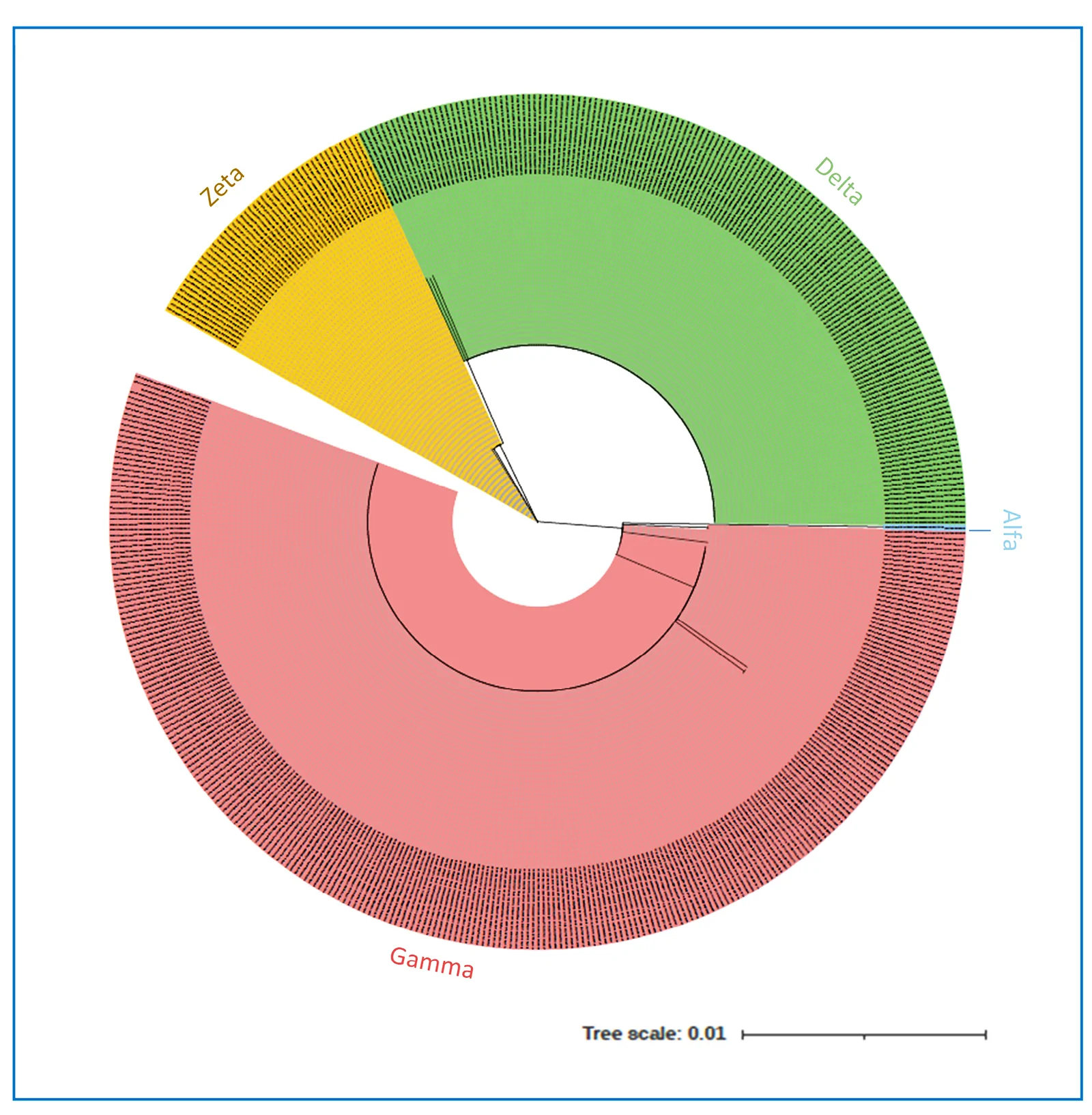
Phylogenetic tree based on the ORF8-N genomic region of SARS-CoV-2 sequences generated in the Federal District, Brazil, during 2021. Within the 2021 epidemiological context of the Federal District, Brazil, the ORF8-N region retained sufficient lineage-discriminatory information to distinguish the main circulating SARS-CoV-2 variants. This restricted target does not provide the same phylogenetic resolution as whole-genome sequencing (WGS).

Continuous *in silico* monitoring was performed throughout the implementation period. WGS was recommended for samples with non-classical mutations or putative non-circulating variants, although this was expected to be necessary for only a small proportion of samples. This strategy was operationally useful as a temporary contingency approach, allowing surveillance to be maintained at scale during periods of NGS unavailability.

The use of alternative methodologies as contingencies to NGS has been crucial, especially in low- and middle-income countries where resources are limited. Sanger sequencing has been used effectively to fill gaps in genomic surveillance, providing timely information on circulating variants^14^. For instance, rapid Sanger sequencing protocols were developed during the COVID-19 pandemic to identify and monitor the spread of variants in Brazil, providing critical insights despite limited NGS capacity^12^. Data from 2021 indicate that the ORF8-N Sanger strategy was operationally informative for that specific epidemiological context. In settings with limited technological resources, the deployment of alternative methodologies can support rapid and effective epidemiological responses. Among these, Sanger sequencing has been highlighted for its adaptability and cost-effectiveness, enabling broader participation of laboratories in genomic surveillance efforts^15^.

During the 2021 surveillance period, implementation of this strategy increased the number of SARS-CoV-2 samples analyzed (Figure 3), thereby helping to sustain surveillance activities during temporary NGS reagent shortages until supply chains were restored. These findings support the operational feasibility of targeted sequencing approaches as temporary alternatives under emergency resource constraints. This experience highlights the importance of resource-efficient contingency strategies to strengthen surveillance resilience during public health emergencies.

**FIGURE 3:**
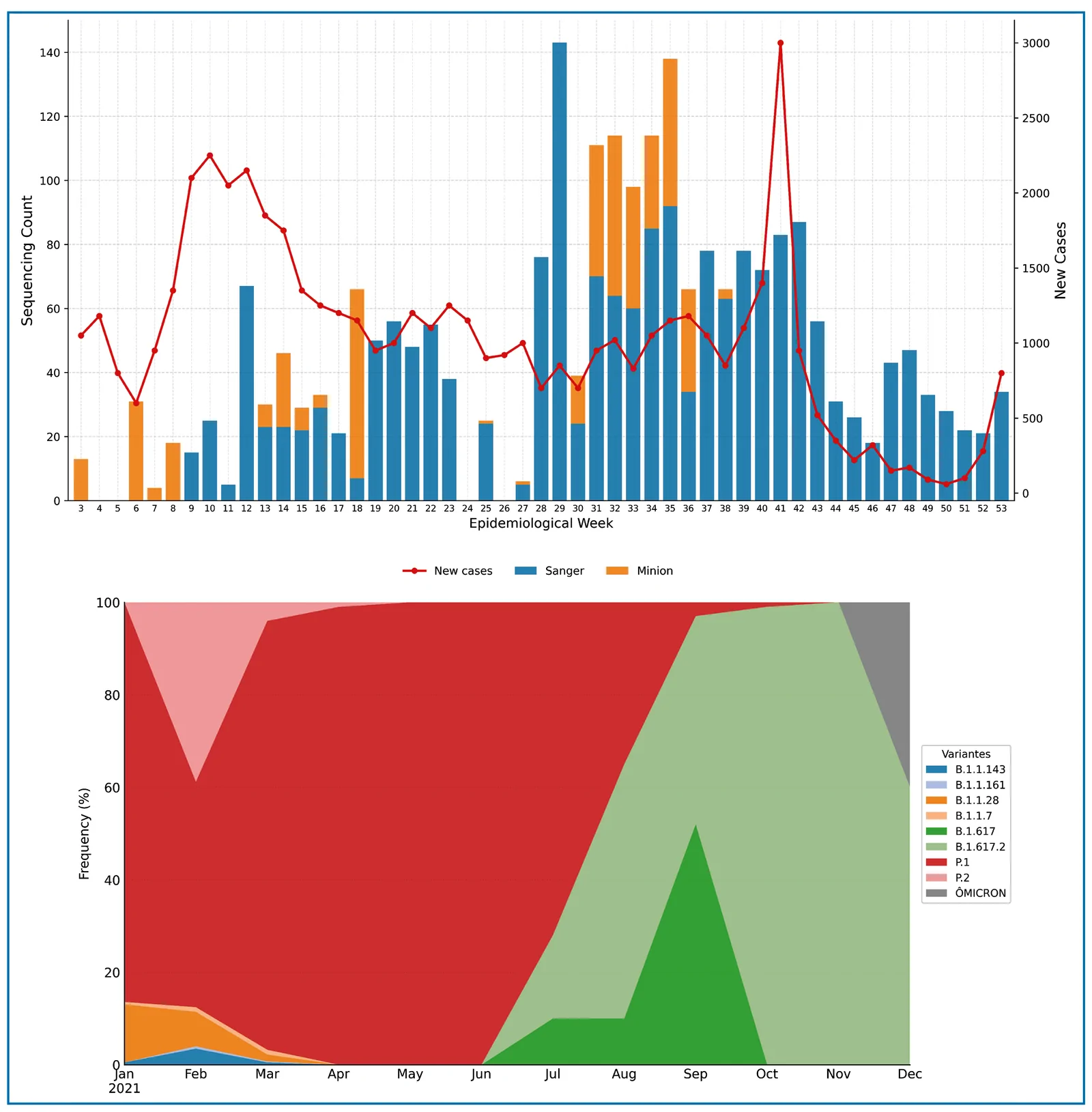
SARS-CoV-2 surveillance activity and variant distribution in the Federal District, Brazil, during 2021. **Upper:** Weekly reported COVID-19 cases and sequencing output by methodology. The red line represents the number of newly reported COVID-19 cases per epidemiological week (right y-axis). Stacked bars indicate the number of samples sequenced using each methodology (left y-axis), with Sanger sequencing shown in blue and MinION sequencing in orange. The x-axis displays epidemiological weeks. **Lower:** Temporal distribution of SARS-CoV-2 variants detected during 2021. The stacked area plot shows the relative frequency (%) of variants identified among sequenced samples from January to December 2021. Each color represents a distinct variant, as indicated in the legend. The x-axis represents calendar months, and the y-axis shows the proportion of each variant relative to the total number of sequenced samples.

Distinct waves of increased COVID-19 cases were identified during the 2021 study period (Figure 3). Following the first wave, the implementation of the Sanger workflow markedly increased the number of samples sequenced. The temporal distribution of variants detected through the contingency workflow was consistent with the epidemiological pattern observed during that period. At least nine distinct SARS-CoV-2 variants were detected in the Federal District of Brazil, including the predominant lineages P.1 and B.1.617.2. The subsequent emergence of the Omicron variant at the end of 2021 preceded the marked increase in COVID-19 cases observed in 2022.

Nevertheless, the landscape of genomic surveillance is dynamic. With the re-establishment of robust NGS supply chains in 2022, the utility of this specific Sanger target declined. Consequently, NGS was reinstated as the primary surveillance tool. These findings highlight that contingency strategies should be temporary and adaptable to both resource availability and viral evolution. This study was not designed as a formal analytical validation study. Instead, the quantitative assessment focused on the overall agreement between WGS-derived lineage assignments and ORF8-N-based lineage grouping. Although the ORF8-N region provided sufficient discriminatory capacity for the 2021 epidemiological context, targeted sequencing inherently offers lower phylogenetic resolution than WGS and may limit the detection of emerging mutations outside the selected region, recombinant variants, or complex evolutionary events. Consequently, this approach should be interpreted as an emergency surveillance contingency strategy rather than a replacement for comprehensive genomic surveillance frameworks.

Notably, this experience highlights the importance of operational preparedness, workforce training, and adaptable laboratory workflows in maintaining genomic surveillance during public health emergencies. The ORF8-N Sanger strategy should be viewed as a context-dependent contingency approach developed during a temporary NGS supply disruption, rather than a substitute for WGS-based surveillance. After the restoration of NGS supply chains and the continued evolution of SARS-CoV-2, comprehensive sequencing again became the preferred strategy for routine genomic surveillance. Nevertheless, this experience reinforces that public health laboratories benefit from maintaining technical capacity and institutional preparedness to implement alternative workflows when standard surveillance strategies are temporarily compromised. Similar contingency approaches were adopted across multiple settings during the COVID-19 pandemic, particularly when sequencing capacity was constrained. Sanger-based workflows were successfully applied in Brazil and other regions as resource-adapted strategies to support variant monitoring when comprehensive genomic sequencing was limited. Collectively, these experiences underscore the importance of developing resilient surveillance capacity and operational flexibility to sustain genomic monitoring during public health emergencies^14^.

## Data Availability

Data available upon request: Research data are available upon reasonable request to the corresponding author. Publicly available SARS-CoV-2 genome sequences analyzed in this study were obtained from the GISAID database. Sequences generated by our laboratory during the study period are available upon request, in accordance with institutional policies and applicable data-sharing agreements.
