# Supplementary material for: Targeted ORF8-N Sanger Sequencing as a SARS-CoV-2 Surveillance Contingency During Supply Shortages
Source: Rev Soc Bras Med Trop. 2026 Sep 14;59:e0591-2025. doi: 10.1590/0037-8682-0591-2025 (PMC13573775; doi:10.1590/0037-8682-0591-2025)
Supplement: Supplementary file 1 [file 1678-9849-rsbmt-59-e0591-2025-md1.pdf]

Revista da Sociedade Brasileira de Medicina Tropical
Journal of the Brazilian Society of Tropical Medicine

Supplementary Table 1. Concordance between phylogenetic clustering and ORF8–N molecular signatures in SARS-CoV-2 genomes from the Federal District, Brazil. Phylogenetic reconstruction of 574 SARS-CoV-2 whole-genome sequences demonstrated complete agreement between lineage designation and ORF8–N molecular signatures. Distinct and lineage-specific profiles were observed for Gamma (P.1), Delta (B.1.617.2), Zeta (P.2), and Alpha (B.1.1.7), with all genomes clustering consistently within their respective phylogenetic groups and no discordant classifications detected.

Lineage / Variant	WGS-derived genomes analyzed (n)	Informative ORF8–N profile (amplicon pattern)	Consistent ORF8–N grouping (n)	Discordant or ambiguous profile (n)	Agreement (%)
**Gamma (P.1)**	264	ORF8/N +	264	0	100
**Delta (B.1.617.2)**	262	ORF8/N +	262	0	100
**Zeta (P.2)**	47	ORF8/N +	47	0	100
**Alpha (B.1.1.7)**	1	ORF8/N +	1	0	100
**Overall**	**574 genomes**	**Distinct lineage-specific molecular signatures**	**574**	**0**	**100**
